# Mitochondrial and cellular mechanisms for managing lipid excess

**DOI:** 10.3389/fphys.2014.00282

**Published:** 2014-07-31

**Authors:** Miguel A. Aon, Niraj Bhatt, Sonia C. Cortassa

**Affiliations:** Division of Cardiology, Department of Medicine, Johns Hopkins University School of MedicineBaltimore, MD, USA

**Keywords:** palmitoyl CoA, lipid droplet, perilipin, beta-oxidation, redox environment, energetics, reactive oxygen species

## Abstract

Current scientific debates center on the impact of lipids and mitochondrial function on diverse aspects of human health, nutrition and disease, among them the association of lipotoxicity with the onset of insulin resistance in skeletal muscle, and with heart dysfunction in obesity and diabetes. Mitochondria play a fundamental role in aging and in prevalent acute or chronic diseases. Lipids are main mitochondrial fuels however these molecules can also behave as uncouplers and inhibitors of oxidative phosphorylation. Knowledge about the functional composition of these contradictory effects and their impact on mitochondrial-cellular energetics/redox status is incomplete. Cells store fatty acids (FAs) as triacylglycerol and package them into cytoplasmic lipid droplets (LDs). New emerging data shows the LD as a highly dynamic storage pool of FAs that can be used for energy reserve. Lipid excess packaging into LDs can be seen as an adaptive response to fulfilling energy supply without hindering mitochondrial or cellular redox status and keeping low concentration of lipotoxic intermediates. Herein we review the mechanisms of action and utilization of lipids by mitochondria reported in liver, heart and skeletal muscle under relevant physiological situations, e.g., exercise. We report on perilipins, a family of proteins that associate with LDs in response to loading of cells with lipids. Evidence showing that in addition to physical contact, mitochondria and LDs exhibit metabolic interactions is presented and discussed. A hypothetical model of channeled lipid utilization by mitochondria is proposed. Direct delivery and channeled processing of lipids in mitochondria could represent a reliable and efficient way to maintain reactive oxygen species (ROS) within levels compatible with signaling while ensuring robust and reliable energy supply.

*Discovery consists of seeing what everybody has seen and thinking what nobody has thought*.

Albert Szent-Gyorgyi

## Introduction

The role of lipids in human health, nutrition, and disease is taking center stage. Several circumstances including hotly debated issues concur to explain this unusual interest. Among them, pressing societal and biomedical issues concerning the epidemic proportions of obesity and related diseases in the United States and its increasing prevalence worldwide. Higher food consumption, decline in physical activity and a progressively aging population are among the social and behavioral roots of this phenomenon. Biologically, it adopts the form of a so-called “metabolic syndrome,” a set of comorbidities including upper body obesity, insulin resistance, dyslipidemia, and hypertension that increase the risk for developing type 2 diabetes, coronary artery disease, and stroke (Kok and Brindley, 2012; Schilling and Mann, 2012).

Functional impairments associated with increased circulating levels of lipids and their induced metabolic alterations in fatty acids (FAs) utilization and intracellular signaling, have been broadly termed lipotoxicity (Wende et al., 2012). Current scientific debates concern the association of lipotoxicity with the onset of insulin resistance in skeletal muscle, and with heart dysfunction in obese and diabetic patients.

Mitochondrial function is closely associated with the mounting attention on lipids. One obvious reason is that mitochondria are the main site of lipid degradation. However, the major driving force underlying this association is the fundamental role played by mitochondrial dysfunction in aging and acute or chronic disease conditions such as metabolic disorders (obesity, diabetes), cancer, inflammatory disorders, neurodegeneration, and cardiovascular disease (Akar et al., 2005; Aon et al., 2009; Bugger and Abel, 2010; Camara et al., 2011; Martinez-Outschoorn et al., 2012; Wallace, 2012; Helguera et al., 2013; Cortassa et al., 2014; Rossignol and Frye, 2014).

**Figure 1:**
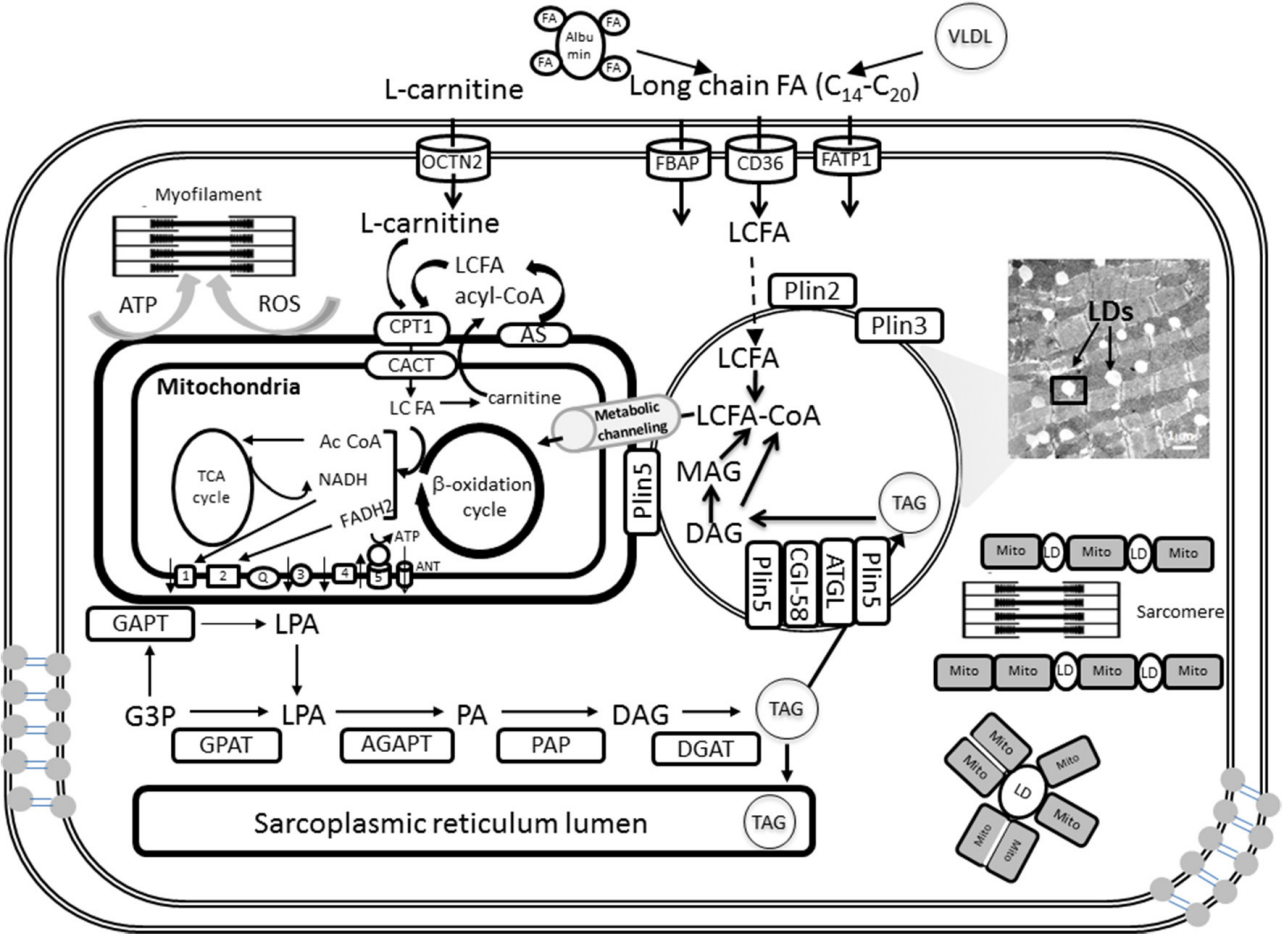
**Triglyceride synthesis, storage in lipid droplets, and FA oxidation in cardiomyocyte mitochondria**. A detailed explanation of the processes depicted in this figure will be found in sections Lipid Droplets and TAG Metabolism and Fatty Acids and Mitochondrial Function of the main text. LDs can be intercalated with mitochondria or surrounded by them as shown schematically at the right bottom. When mitochondria and LD interact in close contact the scheme suggests that FA degradation and activation occur at the contact sites between both organelles. FA precursors of β-oxidation will be subsequently metabolically channeled to the matrix, and likely β-oxidation, through known pathways (see section Metabolic Channeling of Lipid Utilization From Close Contacts Between Mitochondria and Lipid Droplets: A Hypothetical-Qualitative Model in the text for more details).

Cells protect themselves from lipotoxicity or death (Bernardi et al., 2002; Penzo et al., 2002) by either oxidizing FAs or sequestering them as triacylglycerol (TAG) within lipid droplets (LDs) (Greenberg et al., 2011; Walther and Farese, 2012) (Figure 1). The ability to store TAG in LDs is evolutionarily conserved and observed in yeast, plants, invertebrates, and vertebrates (Walther and Farese, 2012). LDs constitute a highly dynamic FA storage pool that can be used for energy reserve. Recent evidence shows that acute exercise can trigger changes in the dynamics of LD assembly, morphology, localization and mobilization in skeletal muscle, a process regulated by a broad genetic program affecting the spatial and metabolic interaction between mitochondria and LDs. In this process, the exercise-responsive transcriptional coactivator PGC-1α appears to play a key role in coordinating intramuscular LD programming with mitochondrial remodeling (Koves et al., 2013).

There is abundant anecdotal evidence describing close interaction between mitochondria and LD. Early observations indicated that mitochondria are often located near a supply of substrate, or at sites in the cell known to require the ATP generated by the mitochondrion (Lehninger, 1965). Occasional close associations between mitochondria and LDs were found in a variety of tissues such as myocardium, liver, pancreas, and brown adipose. As described by Ghadially (1997):

“… A single mitochondrion may appear close to, spread out over, or fused to the surface of a small LD, or several mitochondria may be seen surrounding a larger LD. In other instances, the LD may lie in a deep invagination of the mitochondrial envelope, and it is clear that in another plane of sectioning such a droplet could easily be mistaken for a lipid inclusion in the mitochondrion…, particularly if the invaginating membranes are not visualized.”

As early as 1958, Palade and Schidlowski suggested that these close associations were meaningful because they “*bring the mitochondrial enzymes into close contact with the lipidic substrate*” (Palade and Schidlowski, 1958, quoted by Ghadially, 1997). Although potential artifacts from sample preparation cannot be ruled out, and that pathologically altered mitochondria can have an influence, when describing lipidic inclusions in mitochondria, Ghadially (1997) wrote:

“… lipidic inclusions were noted in normal-looking mitochondria with well-formed cristae, where presumably the lipid has a physiological role.”

More recent experimental data puts on a more solid ground the idea that there are both physical and metabolic interactions between LD and mitochondria. These interactions appear to be modulated by relevant physiological situations such as fasting and exercise training. Available evidence also shows that proteins located in the LD surface closely interact with enzymes of the lypolytic cascade modulating FA acid efflux from the droplet.

## Lipid droplets and TAG metabolism

TAG is the major form of energy storage that with sterol esters serve as reservoirs of membrane lipid components (Walther and Farese, 2009). In cardiomyocytes TAGs are synthesized by acyltransferases and phosphatases at the sarcoplasmic reticulum and mitochondrial membrane and then packaged into LDs (Walther and Farese, 2009; Singh and Cuervo, 2012; Kienesberger et al., 2013). TAG synthesis is initiated by glycerol-3-phosphate acyltransferases (GPAT) at the mitochondrial and sarcoplasmic reticulum membrane and then completed at the sarcoplasmic reticulum by sn-1-acyl-glycerol-3-phosphate acyltransferase (AGPAT), phosphatidic acid phosphatase (PAP), and sn-1,2-diacylglycerol acyltransferase (DGAT) reactions (Kienesberger et al., 2013) (Figure 1). Newly formed TAGs are packaged into cytoplasmic LDs. Thus, lipids are not stored as FAs but as TAGs (triglycerides) produced by a series of esterification reactions that combine three FA molecules with glycerol 3-phosphate; for example, the TAG for palmitate is tripalmitin.

LDs are considered dynamic cellular organelles rather than simple lipid storage depots that, relatively recently, have been implicated in many biological processes (Walther and Farese, 2009, 2012; Greenberg and Coleman, 2011; Singh and Cuervo, 2012). LDs size varies from a diameter of 0.1 μm in yeast to over 100 μm in a white adipocyte. LDs consist of a single protein-decorated phospholipid monolayer that delimits their hydrophobic core from the rest of the cell (Fujimoto and Parton, 2011). The hydrophobic core contains neutral lipids, most notably TAG and sterol esters. The adipose tissue LD has a core predominantly formed by TAG whereas in most cells cholesterol and TAG share the nuclear core of the LD (Singh and Cuervo, 2012). LDs are prominent in many types of mammalian cells, with adipocytes being the most highly specialized for lipid and energy storage. LDs interact with the endoplasmic reticulum and the mitochondria—the two organelles that have been proposed as sites of formation of the autophagosome limiting membrane (Fujimoto et al., 2008; Murphy et al., 2009; Singh and Cuervo, 2012). Such contact zones are also sites of active lipid synthesis enriched in Acyl CoA:diacylglycerol acyltransferase 2 (DGAT2), the major enzyme catalyzing TAG synthesis (Cases et al., 2001; Walther and Farese, 2009).

TAG stored in LDs is catabolized by the sequential hydrolysis of ester bonds between FAs and the glycerol backbone. TAG hydrolysis is a tightly regulated process that involves a complex interaction between lipases and regulatory proteins (Lass et al., 2011). TAG catabolism is performed by a cascade of lipolytic reactions that is initiated by adipose triglyceride lipase (ATGL) producing diacylglycerol (DAG). Hormone-sensitive lipase (HSL) and monoacylglycerol lipase (MGL) complete the lipolytic cascade by sequentially hydrolyzing DAG and monoacylglycerol (MAG), respectively, (Figure 1). MAG lipase (MGL) performs the final step in TAG catabolism by hydrolyzing MAGs to glycerol and FAs (Kienesberger et al., 2013). The rate of lipolysis can be dramatically stimulated by adrenergic hormones via activation of protein kinase A (PKA). PKA phosphorylates perilipin and HSL and causes a complex set of events leading to TAG hydrolysis.

The FAs released during TAG catabolism are mainly used for β-oxidation and subsequent ATP synthesis via OxPhos in mitochondria (Figure 1; see below: *Fatty Acids and Mitochondrial Function*). In oxidative tissues such as the heart, TAG-derived FAs are utilized as an energy source, but they also serve as signaling molecules as well as building blocks for membranes and complex lipids.

Hepatocytes, heart and skeletal myocytes, adrenocortical cells, enterocytes, and macrophages may all contain large amounts of LDs. Excessive LD accumulation is a hallmark of T2DM, obesity, atherosclerosis, hepatic steatosis, and other metabolic diseases. However, in certain organs like skeletal muscle, intramyocellular triacylglycerol (IMTG) accumulation is not strictly a pathologic phenomenon (see below: *Mitochondria, Lipids and Insulin Resistance*). Lipid content is elevated in red compared with white skeletal muscles and increases in response to habitual exercise in both oxidative and glycolytic fibers. The “athlete paradox” consists of IMTG accumulation observed in endurance-trained athletes that retain insulin sensitivity irrespective of the fact that in some cases IMTGs exceed those measured in sedentary obese or T2DM obese patients (Goodpaster et al., 2001; van Loon et al., 2003; Shaw et al., 2010; Egan and Zierath, 2013; Koves et al., 2013). As with aerobic exercise, both muscle glycogen and IMTG contribute to energy provision during resistance exercise (Koopman et al., 2006).

## Mitochondria and perilipins

The protein family of perilipins (Plin) is associated with LDs. As scaffolding proteins perilipins affect the spatial and metabolic interactions between LD and mitochondria (Figure 1). Development of tissue lipotoxicity and dysfunction is linked to alterations in LD biogenesis and regulation of TAG hydrolysis (Wang and Sztalryd, 2011). Since in response to lipid loading of cells perilipins associate with LDs the role of these proteins is under intense scrutiny.

The Plin protein family, or PAT for perilipin/ADRP/TIP47, is constituted by Plin1 to Plin5, and droplets may contain various combinations of them (Greenberg et al., 2011). Plin1 is the most abundant PAT protein in adipocytes and Plin2 in the liver, where it has been linked to hepatic steatosis. Whereas Plin1 and 4 are limited to adipose tissue, Plin2 and 3 are ubiquitous. Plin1 and 2 are always found in an LD-bound state whereas Plin3 to 5 can be either LD-bound or free in the cytoplasm.

Genetic manipulations aiming at ablating perilipins to infer about their physiological roles and impact on fat deposition have been performed. *Plin1*-null mice are lean and develop systemic insulin resistance as they grow older. *Plin1*-null adipocytes exhibited enhanced rates of constitutive (unstimulated) lipolysis and reduced catecholamine-stimulated lipolysis (Tansey et al., 2001). Together, these data suggested that Plin1 protein enhances catecholamine-stimulated lipolysis and, importantly, that a reduction in Plin1 protein expression is associated with increased constitutive lipolysis, which can promote systemic insulin resistance (Greenberg et al., 2011).

Plin5 is found primarily in oxidative tissues, e.g. skeletal and heart muscles, liver (Bickel et al., 2009). *Plin5* knockout mice lacked detectable LDs in the heart and had significantly reduced myocardial TAG content, an effect that was rescued by lipase inhibition (Kuramoto et al., 2012). The excessive TAG catabolism exhibited by Plin5-deficient hearts was paralleled by increased FA oxidation (FAO) and enhanced ROS levels that led to an age-dependent decline in heart function. Thus, it was suggested that uncontrolled lipolysis and defective TAG storage impair cardiac function through chronic mitochondrial FA overload. Plin5 may regulate LD degradation and the flux of lipolysis-derived FAs to mitochondria for energy production (Figure 1) (Kienesberger et al., 2013). Plin5 overexpression in cardiac muscle produced a robust increase in LDs resulting in cardiac steatosis but without major consequences for heart function. This data indicated that Plin5 plays a critical role in droplet formation and stabilization via its regulatory role of lipolysis *in vivo* (Wang et al., 2013). Interestingly, mitochondria in heart tissue from the Plin5 overexpressor appeared to always be distributed in tight clusters around LDs exhibiting a significant increase in size without changes in number as revealed by morphometric analysis (Wang et al., 2013). In skeletal muscle, Plin5 overexpression increased IMCL content without hindering insulin mediated glucose uptake while promoting the expression of genes involved in mitochondrial FAO and fat catabolism (Bosma et al., 2013).

In liver, down-modulation of Plin2 promotes a reduction in hepatic steatosis and increases insulin sensitivity, but a reduction in both Plin2 and Plin3 causes insulin resistance (Greenberg et al., 2011). In the heart, Plin2 does not promote the interaction of mitochondria with LDs, but increased TAG accumulation associated with reduced presence of ATGL in LD and decreased lipolysis (Wang et al., 2011). As the first enzyme from the lipolytic cascade (Zimmermann et al., 2004), the constitutive activity of ATGL is predominantly responsible for basal levels of lipolysis (Greenberg et al., 2011). ATGL overexpression in a cardiomyocyte-specific manner decreased myocardial TAG and lipotoxic intermediates accumulation in type 1 diabetic mice (Pulinilkunnil et al., 2013). This resulted in decreased reliance on FAO, and preserved content of respiratory complexes as well as cardiac function during early stages of diabetes.

Overall, the reported data indicate that reduced expression of perilipins may promote both lipolysis and fat oxidation, resulting in reduced intracellular TAG and adipose mass. On the other hand, excessive lypolysis and defective lipid storage may promote insulin resistance and impaired cardiac function through chronic mitochondrial FA overload. Consequently, lipid storage and utilization appears to be a tightly regulated cellular process.

## Fatty acids and mitochondrial function

Preservation of the intracellular redox environment (RE) is crucial for vital functions such as division, differentiation, contractile work and survival amongst others (Schafer and Buettner, 2001; Aon et al., 2007, 2009; Brown et al., 2010; Fisher-Wellman and Neufer, 2012; Jeong et al., 2012; Lloyd et al., 2012; Muoio and Neufer, 2012; Aggarwal and Makielski, 2013). Mitochondria are main drivers of the intracellular RE (Aon et al., 2010, 2012; Stanley et al., 2011; Tocchetti et al., 2012; Fisher-Wellman et al., 2013; Kembro et al., 2013) and together with peroxisomes constitute the main subcellular compartments where lipid degradation occurs. Yet, the impact of lipids on mitochondrial redox status and ROS emission, and their links to energetics are not fully elucidated.

FAs are main metabolic fuels in heart and skeletal muscle, and β-oxidation represents their main degradation pathway. The rate of β-oxidation is led by demand since an increase in work rate and ATP utilization leads to faster oxidative phosphorylation (OxPhos) and tricarboxylic acid (TCA) cycle activity. In turn, the decrease in NADH and acetyl-CoA (AcCoA) levels leads to an increase of the β-oxidation flux (Neely et al., 1969; Oram et al., 1973; Eaton et al., 1996a; Eaton, 2002; Lopaschuk et al., 2010).

Lipids are supplied in the form of albumin-bound FAs secreted from adipose tissue or by catabolism of very low density lipoprotein (VLDL) complex by coronary vascular endothelial lipoprotein lipases (Figure 1). Long chain FA (LCFA) transport requires carrier proteins in the sarcolemma (FATP1, fatty acid transporter protein 1; FABP, plasma membrane-associated fatty acid-binding protein; LCFAT, long-chain fatty acid transporter; OCTN2, plasma membrane sodium-dependent carnitine transporter; FAT/CD36, fatty acid translocase CD36) and the mitochondria (CPT1, carnitine palmitoyltransferase 1; CACT, carnitine:acylcarnitine translocase).

Upon entry into the cell, LCFA first gets activated by forming thioesters with coenzyme A (CoA), LCFA-CoA, and is either oxidized in the mitochondria via β-oxidation or forms TAG by esterification (Figure 1). Subsequently TAGs can be stored in the form of LD. Long-chain FAs are activated on the mitochondrial outer membrane by the long-chain acyl-CoA synthetase but the mitochondrial inner membrane is not permeable to these acyl-CoAs. CPT1 catalyzes the conversion of long-chain acyl CoA to long-chain acylcarnitine, which is subsequently shuttled into the mitochondria (Lopaschuk et al., 2010). Control at the level of CPT1 activity appears to be important in heart and skeletal muscle β-oxidation flux (Awan and Saggerson, 1993; Lopaschuk et al., 1994; Zammit, 1999; Eaton, 2002).

After its formation by CPT1, the long-chain acylcarnitine is translocated across the inner mitochondrial membrane by CACT that involves the exchange of carnitine for acylcarnitine. CACT has extremely high activity in most cell types with active β-oxidation (Ramsay and Tubbs, 1976; Noel et al., 1985; Eaton, 2002). CACT is a critical step in the translocation of FA moieties into the mitochondria, as evidenced by the development of cardiomyopathies and irregular heartbeats in individuals with CACT deficiencies (Lopaschuk et al., 1994, 2010).

In the matrix, acylcarnitine is converted back to acyl CoA and catabolized via β-oxidation. The β-oxidation of activated FAs occurs within the mitochondrial matrix and is catalyzed by the sequential action of four enzyme families (acyl-CoA dehydrogenase, enoyl-CoA hydratase, 3-hydroxyacyl-CoA dehydrogenase, and 3-ketoacyl- CoA thiolase), with acyl-CoA dehydrogenase exhibiting different substrate specificity for short-, medium-, long- and very long-chain acyl-CoAs (Kunau et al., 1995; Eaton et al., 1996a; Kerner and Hoppel, 2000). The end product of each cycle of β-oxidation is AcCoA, shortening the LCFA by 2 carbons. Ac CoA then enters the TCA cycle for complete oxidation rendering reducing equivalents in the form of the electron donors NADH and FADH_2_ leading to ATP synthesis via OxPhos in the respiratory chain (Figure 1). Ultimately, ATP is utilized by the contractile machinery to transduce chemical energy into mechanical work. ROS may also affect contractile performance via signaling or redox modification of sensitive cysteines from, e.g., myosin heavy chain (Canton et al., 2011; Steinberg, 2013).

Besides their metabolic role in the provision of energy, long-chain free FAs exert diverse effects on cellular membranes and on the catalytic activities of many enzymes (Loskovich et al., 2005). FAs play the dual role of uncouplers and inhibitors of mitochondrial respiration (Wojtczak and Schonfeld, 1993) through a protonophoric effect on the inner membrane, and an inhibitory action on the electron transfer chain (Schonfeld and Reiser, 2006; Schonfeld and Wojtczak, 2007, 2008). Additionally, FAs have the potential to drastically alter mitochondrial membranes permeability through opening of the permeability transition pore (Scorrano et al., 2001; Bernardi et al., 2002; Penzo et al., 2002, 2004). Excluded from these effects are the acyl-CoAs that do not exert protonophoric activity and do not uncouple OxPhos because they are unable to cross the inner mitochondrial membrane (Wojtczak, 1976).

Free FAs can act as specific complex I-directed inhibitors (Loskovich et al., 2005; Schonfeld and Wojtczak, 2008), and long-chain acyl-CoAs are known inhibitors of ANT (Pande and Blanchaer, 1971; Lerner et al., 1972; Wojtczak, 1976). The inhibition is of a competitive character (Duszynski and Wojtczak, 1975) and strongly depends on the carbon chain length of the fatty acyl moiety (Morel et al., 1974). Further evidence that FAs, in their anionic form, can be substrates for transport by ANT was given by their inhibitory effect on ATP and ADP exchanges (Wojtczak and Zaluska, 1967; Schonfeld et al., 1996; Klingenberg, 2008). According to the FA cycling model (Skulachev, 1991) undissociated FA molecules can undergo a spontaneous flip-flop from the outer to the inner leaflet of the inner mitochondrial membrane where they release protons because of the alkaline milieu of the matrix. Then, in the form of anions, they are transported back to the external leaflet by ANT; one proton is transferred from the external space to the matrix compartment per molecule of the FA per cycle. In this manner, FAs can lead to energy dissipation through a selective protonophoric action mediated by coupling of transmembrane movement of the fatty acyl anion (via the ANT, uncoupling proteins, UCPs, and/or other inner membrane carriers). These events result in dissipative proton cycling that decreases the proton motive force thereby affecting respiration, ATP synthesis, and ion homeostasis.

Palmitoyl CoA inhibits the ANT independently from β-oxidation, according to more recent evidence obtained in isolated mitochondria from rat liver (Ciapaite et al., 2005) and guinea pig heart (Aon and Cortassa, unpublished) respiring on G/M. In the case of liver mitochondria it was shown that the ANT inhibition induced changes in intra- and extra-mitochondrial ATP concentrations and ΔΨ_m_. This interference with the ANT carrier increased ΔΨ_m_ and the reduction level of coenzyme Q (Bakker et al., 2000) both expected to promote the formation of ROS. Studies further showed that the PCoA-elicited concentration-dependent H_2_O_2_ formation can be explained by its effect on ΔΨ_m_ that in the presence of 5 μM PCoA showed a 13 mV increase (Ciapaite et al., 2006). The specific action of PCoA on the ANT in the liver (Ciapaite et al., 2006), is in contrast with an apparent multi target effect in the heart (Aon and Cortassa, unpublished). These differences may be given by intrinsic functional differences due to species (rat, guinea pig) or organ specificity, e.g., liver and heart mitochondria. Differences may also be linked to the presence of distinct FA transporters (FATPs or SLC27As) or FA binding proteins (FABPs).

## Mitochondria, lipids, and insulin resistance

The shift from intermediate values of RE, corresponding to ROS levels compatible with signaling functions (Aon et al., 2010; Cortassa et al., 2014), toward either more reducing or oxidizing conditions is a topic of great potential importance and interest with implications for insulin signaling. Indeed, the association between lipotoxicity and the onset of insulin resistance in skeletal muscle is a hotly debated subject (Muoio and Neufer, 2012). One side posits that it is due to dysfunctional mitochondria with intrinsic deficiencies in OxPhos and deficits in fat oxidation. These impairments impinge on insulin signaling by diverting FAs away from oxidation and toward production of DAGs, ceramide and other toxic lipid species (Lowell and Shulman, 2005; Roden, 2005). The other side of the debate notes that this idea is incompatible with the principles of bioenergetics because mitochondrial respiration is governed by energy demand; intracellular lipids will accumulate whenever FAs supply exceeds the energy needs of the cell. Consequently, they suggest that the etiology of muscle insulin resistance is grounded on the fundamental principles that govern cellular and mitochondrial bioenergetics and the redox stress that is placed on the respiratory system when energy supply persistently outpaces energy demand (Muoio and Neufer, 2012). In agreement with this idea other authors have emphasized that the matching between increased FA availability and oxidative capacity distinguishes the increase in IMTG following endurance training from obesity/diabetic conditions. Chronic exercise training can elicit high oxidative capacity conferred by higher mitochondrial content but not mitochondrial function. Under these conditions, lipid infusion in endurance-trained athletes is able to reduce insulin sensitivity only by 29% as compared to 63% in untrained subjects (Phielix et al., 2012).

Whereas in exercise training IMTG reflects an increased reliance on fats as substrate, in obesity/diabetes will imply accumulation of lipid metabolites [long chain fatty acyl-CoA (LCFA-CoA), DAG, and ceramide] that are responsible for the impairment in insulin action rather than the IMTG pool contained in LDs (Schrauwen et al., 2010; Fisher-Wellman and Neufer, 2012). Apparently, increased concentrations of intramuscular LCFA-CoA and DAG activate PKC, which appears to induce impairments in insulin signaling via serine phosphorylation of the insulin receptor substrate-1. In a model of diet-induced obesity, accumulation of acylcarnitines, as products of incomplete β-oxidation, was shown in skeletal muscle (Koves et al., 2008). These findings led to the idea of a mitochondria-derived signal that couples incomplete β-oxidation with insulin resistance. Chronic elevations of incomplete oxidation intermediates of FAs and branched-chain amino acids (Newgard, 2012) might foster a mitochondrial microenvironment that is conducive to higher H_2_O_2_ release from mitochondria with potential to modulate insulin signaling (Fisher-Wellman and Neufer, 2012; Muoio and Neufer, 2012).

The debate about the role of mitochondrial and lipid metabolism at the origin of insulin resistance is highly relevant for the diabetic heart because of its heavy dependence on fats for function (Holloway et al., 2009, 2011). The debate centered on the mitochondrial load-oxidative potential in skeletal muscle, is also relevant for the heart where function is led by energy demand. In fact, lipid accumulation in the heart is largely seen as a mismatch between supply and demand, i.e., lipids amass when supply outpaces demand.

A fundamentally important question still heavily debated is whether or not a shift in substrate preference toward fat oxidation lowers disease risk (Muoio and Neufer, 2012). FAs and glucose are the two major fuels driving heart contraction. In type 2 diabetes and obesity FAO is increased (Lopaschuk, 2002; Carley and Severson, 2005) but our knowledge about the combined effects of hyperglycemia, a hallmark of diabetes, and high FA availability, on metabolism, redox/ROS balance and their impact on heart function is incomplete. Although the healthy heart is flexible regarding fuel selection, in the metabolically challenged diabetic heart by high levels of glucose and fat, the factors contributing to dysfunction and which are beneficial as energy source or redox donors are still unclear. Existing compelling evidence indicates that substrate-driven redox status plays a critical role in cardiac contractile performance in type 2 diabetes where cellular/mitochondrial redox and energetics are altered (see below: *Mitochondrial, Cellular and Organ Mechanisms for Managing Lipid Affluence*) (Anderson et al., 2009a; Tocchetti et al., 2012). Overall, there is no disputing that lipid oxidation confers a metabolic advantage during starvation and exercise, but the role of fuel selection *per se* in defending against metabolic disease needs further investigation.

## Mitochondrial, cellular, and organ mechanisms for managing lipid affluence

As important fuels of cellular function it is very well known how FAs are degraded by mitochondria. Yet, the mechanisms by which mitochondria manage lipid excess are largely unknown. The role of β-oxidation *per se* as an underlying cause of obesity-associated glucose intolerance remains a topic of active research and debate (Fisher-Wellman and Neufer, 2012; Muoio and Neufer, 2012). Furthermore, mitochondria play a central role in the development of diabetes and obesity complications (Bugger and Abel, 2010; Sivitz and Yorek, 2010) and their energetic/redox dysfunction is directly involved in the redox imbalance exhibited by the heart (Tocchetti et al., 2012; Frasier et al., 2013) and skeletal muscle (Anderson et al., 2009a).

Mitochondria and lipid oxidation play a predominant role as drivers of the intracellular RE. FAs are a major source of cellular ATP which, in the heart, is synthesized up to two thirds via reducing equivalents (e.g., 24 NADH, 8 FADH_2_ for palmitate) derived from β-oxidation in mitochondria. The higher energetic budget provided by the saturated FA palmitate (three times higher than from glucose when ATP/mol substrate is considered) in the form of reducing power provides electrons to antioxidant systems and the mitochondria respiratory/energetic machinery. In agreement with the prominent role of lipids on the intracellular redox status, it was shown that Palm determined a transition from oxidized-to-reduced cellular redox status in cardiomyocytes from type-2 diabetic (*db/db*) hearts abating ROS levels drastically (Tocchetti et al., 2012). This effect was coupled to a marked GSH rise both in wild type and *db/db* myocytes. As a consequence of its favorable effect on cellular redox balance, Palm significantly improved isoproterenol-induced contractile reserve in *db/db* cardiomyocytes (Tocchetti et al., 2012).

Keeping a proper cellular/mitochondrial RE is vital for optimal excitation-contraction (EC) coupling as well as energy supply in the heart (Burgoyne et al., 2012; Christians and Benjamin, 2012; Nickel et al., 2013, 2014). Intracellular redox balance affects Ca^2+^ handling by interfering with a wide range of proteins implicated in EC coupling (Fauconnier et al., 2007) including the SR Ca^2+^ release channels [the ryanodine receptors], the SR Ca^2+^ pumps, and the sarcolemmal Na^+^/Ca^2+^ exchanger (Zima and Blatter, 2006; Dedkova and Blatter, 2008). Also unknown is whether the mechanisms utilized by mitochondria to deal with lipid excess differ between organs. Important examples are the skeletal and cardiac muscles where β-oxidation predominates due to their lack of *de novo* lipogenesis (Eaton, 2002). Certainly, the organ's functional specificity plays a role. As a matter of fact, skeletal muscle is the largest glycogen storage organ (~4-fold the capacity of the liver) thus critical for glycemic control as the predominant (~80%) site of glucose disposal under insulin-stimulated conditions (DeFronzo et al., 1981; Egan and Zierath, 2013). On the other hand, the heart carries out its pump function transducing the chemical energy stored in FAs and glucose into mechanical and electrical energy. At rest, the heart cycles about 6 kg of ATP every day while beating about 100,000 times (Neubauer, 2007). Mitochondria provide the bulk of the ATP needed for cardiac muscle contraction (about two thirds) and sarcolemmal and sarcoplasmic ion transport (one third), responsible for the Ca^2+^ transients and electrical activity in cardiac cells (Solaini and Harris, 2005; Cortassa et al., 2009; Nickel et al., 2013).

The far higher amounts of O_2_ processed by the heart on a specific basis with respect to, e.g., brain and skeletal muscle (Rolfe and Brown, 1997), and its continuous activity, make this organ susceptible to oxidative damage (Burgoyne et al., 2012; Christians and Benjamin, 2012). As a matter of fact, myocardial function and the ability of the heart to tolerate stress decline with age (Lakatta and Sollott, 2002). Although the mechanisms contributing to age-related alterations in myocardial function are not fully understood, mitochondrial dysfunction, oxidative stress and the accumulation of oxidant-induced damage are major factors (Fannin et al., 1999; Suh et al., 2003; Judge et al., 2005).

Defects in mitochondrial FA β-oxidation lead to several well-known metabolic disorders, such as Reye syndrome, cardiomyopathy and sudden infant death syndrome (Roe and Ding, 2001; Yang et al., 2001). The maintenance of high levels of mitochondrial β-oxidation could reduce the excessive fat accumulation and storage leading to human obesity. Lipid overload involving TAG accumulation in non-adipose tissues characterizes disorders such as hyperlipidemia and lipodystrophies, heart dysfunction, liver disease, in both humans and in animal models of obesity and diabetes.

It is becoming increasingly clear that adequate regulation of TAG metabolism in different organs is critical for both energy metabolism and function. Liver and heart respond to the massive influx of lipids from blood by up regulating LD biogenesis, as a mechanism of defense against the toxicity of FAs, which upon esterification get converted into TAG and stored into LD (Lass et al., 2011). Failure to do so in the liver originates pathogenic conditions such as steatosis and steatohepatitis (Greenberg et al., 2011). The lipid excess situation is also relevant for heart function in T2DM where FAs are preferred fuels (Lopaschuk et al., 2010). However, under acute, non-chronic, conditions FAs can exhibit advantageous actions, especially in the heart under diabetic conditions (Tocchetti et al., 2012). Cellular TAG accumulation in LDs may be beneficial rather than detrimental because it diverts FAs from pathways leading to cytotoxicity thus serving as a buffer against lipotoxicity (Listenberger et al., 2003).

From the examples and arguments above, it is clear that lipids have a considerable impact on many cellular processes, including mitochondria. This impact influences the functional outcome of several organs such as the liver, skeletal and cardiac muscles. Deregulation of lipid metabolism produces overload that is at the origin or as an aggravating consequence of many diseases. Consequently, the fundamental as well as practical importance of unraveling the mechanisms by which mitochondria handle lipids excess cannot be overstated. First, at the most basic level, we do not know enough about lipids action on mitochondrial energetic and redox functions. Lipids can act both as uncouplers and inhibitors of OxPhos (Wojtczak and Schonfeld, 1993; Bernardi et al., 2002), and the consequences of these contradictory effects on mitochondrial energetic, redox and signaling functions are just starting to be unraveled (Schonfeld and Wojtczak, 2008). Second, besides being the main site of lipid degradation, mitochondria may be actively modulating the balance between lipid storage and utilization.

In the following sections we explore some of the new emerging mechanisms of lipid storage and utilization by mitochondria at the organelle, cellular and organ level in different physiological settings.

### Close contact mitochondria-lipid droplet

Regular exercise and physical activity are considered cornerstones in the prevention, management, and treatment of numerous chronic conditions, including hypertension, coronary heart disease, obesity, T2DM, and age-related muscle wasting (sarcopenia) (Haskell et al., 2007; Colberg et al., 2010; Egan and Zierath, 2013).

Exercise training enhances mitochondrial biogenesis and performance in skeletal muscle (Irrcher et al., 2003), but not in the heart (Li et al., 2011). Whether the same is true in T2DM hearts is unclear. In electron micrographs LDs can be easily detected in type 2 diabetic (*db/db*) (Boudina et al., 2007) or *ob/ob* (Ge et al., 2012) but not in WT mice hearts. In cells LDs can be readily visualized using the fluorescent FA analog (dodecanoic acid) BODIPY that labels neutral lipids in cytoplasmic droplets (Walther and Farese, 2012).

The occurrence of close contact between mitochondria and LD in the heart is remarkable because of its dependence on mitochondrial energetics preferentially fueled by FAs. More noteworthy though is the fact that these close contacts occur in the T2DM heart, where the dependence on fat fueling is even more prominent (Lopaschuk, 2002; Bugger and Abel, 2010). Interestingly, Plin5 overexpression in heart tissue rendered tight mitochondrial clusters around LDs with mitochondria significantly larger but not higher in number (Wang et al., 2013). The same authors proposed that Plin5 could play a regulatory role in the FA flux from LDs to mitochondria under conditions of increased cellular FA influx (Wang and Sztalryd, 2011). These data also suggest that Plin5 with its role of favoring LD accumulation may act to keep the intracellular levels of FA metabolites (e.g., DAG, ceramide) below lipotoxic amounts (see below: *Metabolic Channeling of Lipid Utilization From Close Contacts Between Mitochondria and Lipid Droplets: A Hypothetical-Qualitative Model*).

In skeletal muscle IMTG accumulates and is actively utilized during exercise (Shaw et al., 2010; Egan and Zierath, 2013; Koves et al., 2013). Endurance exercise training increases mitochondrial content (by size not numbers) for men and women but healthy active women have higher IMTG accumulation compared with men due to greater number rather than size of LDs (Tarnopolsky et al., 2007). Interestingly, this study also reported an increase in the physical contact between mitochondria and IMTGs following endurance exercise training. Rates of whole body fat oxidation and IMTG utilization are determined by factors such as diet, intensity and duration of exercise, and fitness. During acute exercise, the contribution of various metabolic pathways to energy provision is determined by the relative intensity and absolute power output of the exercise bout (Egan and Zierath, 2013). The rate of ATP demand and energy expenditure is determined by the absolute power output whereas the relative exercise intensity influences the relative contributions of carbohydrate oxidation and lipid sources, and circulating (extramuscular) and intramuscular fuel stores, to energy provision. As exercise intensity increases, muscle utilization of circulating free FAs slightly declines, whereas utilization of circulating glucose increases progressively up to near-maximal intensities (van Loon et al., 2001).

IMTG breakdown occurs primarily via HSL and ATGL (Watt and Spriet, 2010). Although IMTGs constitute only a small fraction (~1–2%) of whole-body lipid stores they represent an important fuel source during prolonged (>90 min) but moderate intensity exercise. IMTGs can provide ~25% of total energy however their contribution decreases at either higher or lower intensities of exercise (Romijn et al., 1993; van Loon et al., 2001). Maximal rates of fat oxidation occur at moderate exercise intensities (~ 60% VO_2_ max) (Shaw et al., 2010; Egan and Zierath, 2013). At low-to-moderate exercise intensity, the primary substrates fueling skeletal muscle are glucose, derived from hepatic glycogenolysis (or gluconeogenesis) or oral ingestion, and free FAs released by adipose tissue lipolysis. Prolonged exercise (>60 min) at a fixed intensity increases the energy contribution from lipid oxidation (Egan and Zierath, 2013). IMTG stores can be reduced by ~60% following exercise, predominantly in type I muscle fibers (van Loon et al., 2003; Stellingwerff et al., 2007; Shaw et al., 2010; Egan and Zierath, 2013).

Lipophagy, i.e., the turnover of LDs by autophagy, may occur due to random sequestration of cytosolic material by “in bulk” autophagy. However, when lipophagy is activated in response to a lipid challenge or prolonged starvation, a switch toward the preferential sequestration of LD seems to happen, supporting some level of selectivity in this process (Singh et al., 2009). We suggest that this may also be the case for close contacts mitochondria-LD, and that energy demand may be a main elicitor of the interaction between these two organelles. Consonant with this idea, it has been proposed that LDs assembly in skeletal muscle under exercise training would improve the management of high FA influx enabling a more precisely regulated trafficking of substrate to and from IMTG thus contributing to optimal mitochondrial performance and metabolic flexibility (Koves et al., 2013).

### Lipotoxicity and LD accumulation dynamics

In pathologic states lipotoxicity may occur over time, despite TAG accumulation, when either the cellular capacity for TAG storage is exceeded or when triglyceride pools are hydrolyzed, resulting in increased cellular free FA levels. Thus, the duration and extent of lipid overload may determine if a cell is protected or damaged. Whether mitochondrial energy/redox status can alter the balance LD formation and utilization in the short-term is a question that has not been hitherto addressed.

Studies performed with non-invasive spectroscopic techniques have shown elevated IMCL triglyceride content in the left ventricle (i.e., LV steatosis) of obese and T2DM patients (McGavock et al., 2007; Rijzewijk et al., 2008) but its association with early diastolic dysfunction leading to subsequent systolic dysfunction remains controversial (Anderson et al., 2009b; Lopaschuk et al., 2010). Again, lipids through accumulation of triglycerides are at the center of the controversy. In skeletal (Liu et al., 2007) and cardiac (Ussher et al., 2009) muscle, IMCL accumulation as a result of diet-induced obesity is not at all pathogenic, but may even be protective against obesity-associated maladies.

Previous reports have linked ROS-mediated mitochondrial dysfunction to DAG and ceramide, two main products of lipid degradation (Coen and Goodpaster, 2012). Lipid channeling to mitochondria may represent a mechanism by which concentration build-up of these intermediaries is avoided, especially under high energy demand. Based on these premises, we suggest that temporary lipid storage in LDs does not necessarily represent pathophysiological behavior. On the contrary, it may embody an adaptive response, at least in the short-term thus representing an adaptive strategy of lipids utilization ensuring energy supply without affecting neither mitochondrial nor cellular redox status.

## Redox optimized ROS balance and mitochondrial redox and energetics

Lipid metabolites can damage the respiratory chain leading to impaired energetic transition in mitochondria through their dual effect as uncouplers and inhibitors (Wojtczak and Schonfeld, 1993). Impairment of the key state 4→3 energetic transition can occur via inhibition of ANT or ATPsynthase thereby producing a continuous release of ROS irrespective of ADP addition (Tocchetti et al., 2012).

**Figure 2:**
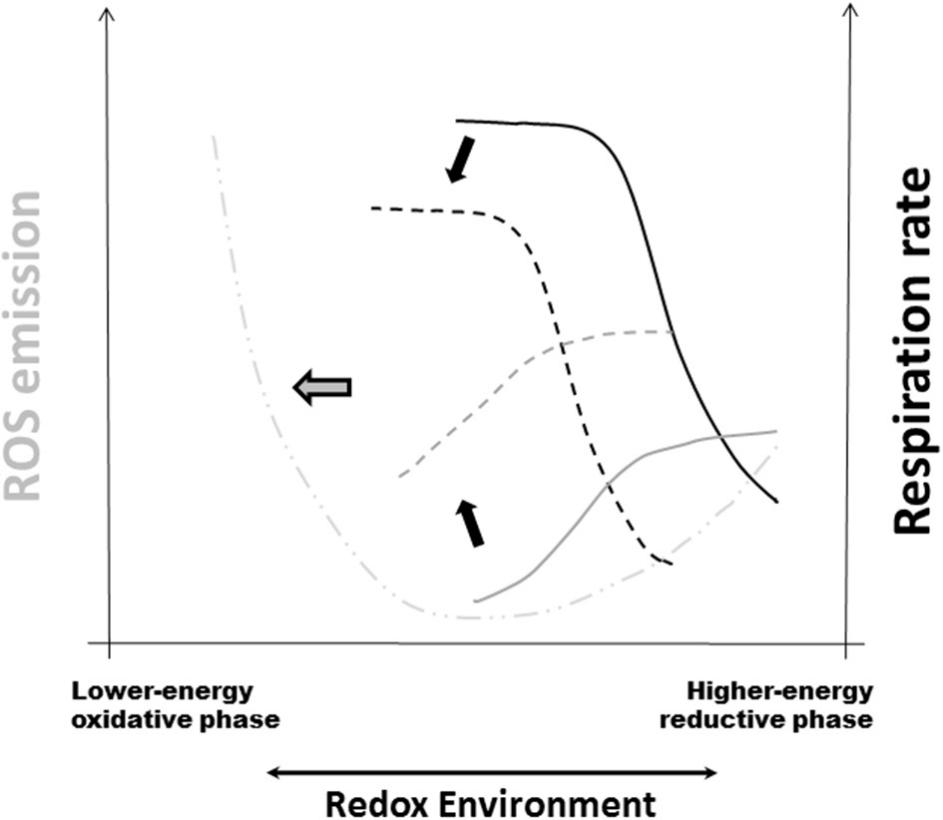
**Redox-Optimized ROS Balance and the effect of oxidative stress on mitochondrial respiration, H_2_O_2_ emission, and the RE**. R-ORB postulates that ROS levels (as the net result of production and scavenging) depend on the intra-cellular and -mitochondrial redox environment (RE). It also proposes that there is a minimum level of ROS emission when mitochondria maximize their energetic output. Under high energy demand, and despite large respiratory rates, ROS emission levels will be kept to a minimum by ROS scavenging systems (Stanley et al., 2011; Aon et al., 2012). Oxidative stress can happen at either extreme of RE, either highly reduced or highly oxidized, but governed by different mechanisms (Aon et al., 2010; Kembro et al., 2014). The plot displays schematically the summary of the response of respiration (black traces) and ROS emission in stressed mitochondria (gray traces) plus further addition of the uncoupler FCCP (dashed-dotted line). Continuous lines correspond to the absence of stress whereas dashed lines belong to mitochondria under stressed conditions (Cortassa et al., 2014). Black arrows indicate the direction of change in VO_2_ and ROS elicited by stress. Notice the shift toward more oxidized RE in the curves corresponding to stressful conditions. The thick gray arrow pointing to the left denotes pathological conditions arising, e.g., from chronic diseases, where severe stress will affect both energetic (e.g., ΔΨ_m_, ADP consumption) and redox [e.g., NAD(P)H, GSH, Trx] functions thus increased mitochondrial ROS emission and higher cytoplasmic ROS levels. Reprinted from Cortassa et al. (2014).

Mitochondria are a main source of ROS but can also be their target. The RE is a major driving force of the crucial energy-redox link of mitochondrial function (Cortassa et al., 2014). The mitochondrial RE depends on the intrinsic redox potential and instantaneous reducing capacity of this organelle as well as its response to the cytoplasmic redox status (Aon et al., 2010; Kembro et al., 2013). In this context, Redox-Optimized ROS Balance (R-ORB) provides a useful conceptual framework to rationalize many results described in the present review. One of the main R-ORB postulates is that ROS efflux from mitochondria will attain a minimum at intermediate values of RE, when VO_2_ reaches a maximum following ADP stimulation (Figure 2) (Cortassa et al., 2014). Under state 3 respiration, glutathione and thioredoxin systems are essential for minimizing ROS release from mitochondria (Aon et al., 2010, 2012; Stanley et al., 2011; Kudin et al., 2012; Cortassa et al., 2014). In excess, lipid precursors of β-oxidation can promote mitochondrial uncoupling and oxidized redox status (Aon and Cortassa, unpublished). In more oxidized RE, away from the optimum (intermediate) RE compatible with minimal ROS, antioxidant systems become overwhelmed leading to pathological ROS overflow (Aon et al., 2010; Cortassa et al., 2014).

Mitochondria function in more oxidative environments in chronic diseases (Tocchetti et al., 2012). Thus, it becomes fundamental to understand how oxidative stress influences the dependence of ROS emission on respiration (Cortassa et al., 2014). When oxidant challenged, mitochondria displayed H_2_O_2_ emission levels 2-fold higher than controls, and exhibited lower respiration (Figure 2). Oxidative stress shifted redox balance toward the more oxidized range where the sensitivity of the ROS efflux to the RE decreases more drastically in state 4 than in state 3 respiration. A 50% decrease in reduced glutathione (GSH) was mainly responsible for the shift of the RE to a more oxidized state (Cortassa et al., 2014).

## Metabolic channeling of lipid utilization from close contacts between mitochondria and lipid droplets: a hypothetical-qualitative model

Recent evidence supports physical and metabolic interactions between LDs and mitochondria mediated by the scaffolding protein Plin 5 (Wang and Sztalryd, 2011; Wang et al., 2011; Koves et al., 2013). Wang and collaborators observed that Plin5-overexpressing cells show decreased LD hydrolysis and palmitate β-oxidation when compared with controls. Instead, palmitate increasingly incorporated into TAGs under basal conditions whereas in protein kinase A-stimulated state LD hydrolysis inhibition was removed and FAs released for β-oxidation. These results suggested that Plin5 regulates LD hydrolysis and controls local FA flux to protect mitochondria against excessive exposure to FA (Wang and Sztalryd, 2011). All these observations are in agreement with the relatively recent realization that the LD proteome is highly dynamic and more complex than previously thought. The LD proteome contains key components of the fat mobilization system and proteins that suggest LD interactions with a variety of cell organelles, including the mitochondria (Beller et al., 2010).

Based on the premise of metabolic links extending beyond physical contact between mitochondria and LDs, we propose a model of metabolic channeling for lipid utilization by mitochondria. According to our model, metabolic channeling represents a way mitochondria can manage lipid affluence in an energetically and redox-controlled fashion. Qualitatively, the lipid utilization channeling model postulates that after TAG degradation, lipids are directly delivered for activation, transport and β-oxidation from the LD to the mitochondrion at the contact site (Figure 1). The model also proposes that β-oxidation may also happen metabolically channeled through the enzymatic components of the lipid degradation pathway organized as a multienzyme complex (Eaton, 2002).

From a structural standpoint, the model is based on direct and close contact between LDs and mitochondria involving their recruitment and surrounding of the LD. The model also postulates membrane fusion-mediated reorganization of intra-mitochondrial membrane and molecular components (Walther and Farese, 2009) as well as lipids segregation within the droplet (Fujimoto and Parton, 2011).

Biochemically, the pathway of long-chain FAO to AcCoA is one of the longest unbranched pathways in metabolism, containing 27 intermediates between palmitoyl-CoA and AcCoA (Eaton, 2002). That the enzymes of β-oxidation may be organized into a multienzyme complex was suggested long ago. In these biomolecular assemblies, sequential catalytic reactions proceed via transfer of the intermediates between individual component enzymes, precluding their diffusion into the bulk aqueous medium, thus “metabolically channeled” (Welch, 1977; Sumegi et al., 1991).

An earlier proposal of metabolic channeling in β-oxidation was based on the detection of low concentrations of intermediates (Garland et al., 1965) and the observation that β-oxidation intermediates that accumulate behaved more like products than intermediates (Stewart et al., 1973; Stanley and Tubbs, 1974, 1975; Eaton et al., 1994, 1996a,b, 1999). This led to the “leaky hosepipe” model for the control of β-oxidation flux (Stewart et al., 1973; Stanley and Tubbs, 1974, 1975) in which channeling of a small, quickly turning-over pool of intermediates was implied (see Eaton, 2002 for a review).

Some aspects of the structural basis for a channeling mechanism in β-oxidation have been described (Ishikawa et al., 2004). Evidence in support of a multifunctional FAO complex within mitochondria, physically associated with respiratory chain supercomplexes that favor metabolic channeling, has been recently reported (Wang et al., 2010). Functionally, the direct delivery of lipids at contact sites, and their channeled processing will avoid elevation of their concentration, thus ruling out the potential inhibitory as well as uncoupling action of FAs (Wojtczak and Schonfeld, 1993). The latter will ensure a reliable and efficient energy supply.

## Concluding remarks

Mitochondria, cells and organs have developed mechanisms that allow managing heavy influx of FAs within functionally reliable limits. The LD as a dynamic storage of FAs can also be seen as a protective mechanism employed by cells to avoid excessive intracellular concentration of FAs thus hindering their potential deleterious effects on mitochondrial function. The tight and reciprocal regulation of lipid storage and utilization is evidenced by genetic manipulation of perilipins indicating that their reduced expression leads to increased lipid oxidation and reduced accumulation of intracellular fat and adipose mass. On the other hand, however, excessive lipolysis and defective lipid storage promotes insulin resistance through mitochondrial FA overload and ROS overflow.

Preservation of the intracellular RE is crucial for vital functions. Mitochondria play a decisive role as the organelle that specifically handles the highest amounts of oxygen processed by the organism thus prone not only to be the source but also the target of oxidative stress. Mitochondrial function needs to sustain energy supply reliably while releasing ROS levels compatible with signaling. However, lipids can derail both of these critical functions. Consequently, the hypothetical lipid utilization channeling model we are proposing herein satisfies the fundamentals of cellular and mitochondrial energetics and redox. In principle, diversion of excess lipids to LDs can be an effective cytoplasmic mechanism for “sequestering” FAs thereby helping to keep low concentration of lipotoxic intermediates resulting from lipid oxidation. Functionally, direct delivery and channeled processing of lipids in mitochondria could represent a reliable and efficient way to ensure energy supply and redox control. Such a mechanism would avoid exceeding the lipid storage capacity thus becoming crucial for skeletal muscle or heart subjected to high workload, and therefore, heavy influx of FAs.

### Conflict of interest statement

The authors declare that the research was conducted in the absence of any commercial or financial relationships that could be construed as a potential conflict of interest.
